# Exploring gender diverse young adults’ gender identity development in online LGBTQIA + communities

**DOI:** 10.1080/26895269.2024.2344534

**Published:** 2024-04-24

**Authors:** Kieran (Kie) Cronesberry, Luke Ward

**Affiliations:** a Northern Region Gender Dysphoria Service, Cumbria, Northumberland, Tyne and Wear NHS Foundation Trust, Newcastle upon Tyne, UK; b Department of Psychology & Sociology, University of Northampton, Northampton, UK

**Keywords:** Gender diversity, identity development, online spaces, transgender, young adults

## Abstract

**Background:**

Online spaces are widely used by gender diverse communities and may reduce the risk of negative psychological outcomes faced by these communities. However, little is known about the role online environments play in the development of gender identity, which may explain the growing number of gender diverse youth ‘coming out’ earlier than seen previously. In this article, gender diverse refers to anyone whose internal sense of gender is incongruent with their assigned sex at birth such as: transgender, non-binary, and agender persons.

**Aim:**

This article examines the role of online lesbian, gay, bisexual, transgender, queer/questioning, intersex, and asexual (LGBTQIA+) communities on gender identity development and examines how the structure of these spaces may positively facilitate this development.

**Methods:**

A sample of 9 gender diverse young adults 19–25 years old participated in online semi-structured interviews which were analyzed using reflexive thematic analysis.

**Results:**

Two main themes were developed: safe spaces in virtual places and the value of online spaces as educational resources.

**Discussion:**

The findings indicate that online LGBTQIA + communities facilitate gender identity development for gender diverse young adults through providing a vital resource of gender diverse specific education and exposure to less visible gender identities. Implications for future research, recommendations for practice with gender diverse youth, and limitations are discussed.

## Introduction

Adolescence is a profound time of change, marked by cultural, social, cognitive, and biological changes (Kinghorn et al., 2018) and development of a sense of identity about who they are, their place in the world, and seeking a community of likeminded peers (McDonagh et al., 2018; Sawyer et al., 2018). Historical literature on identity development uses a cisgenderist lens (Kohlberg, 1966), or focuses on sexual identity development (Cass, 1979). There remains a gap in our understanding in how people in gender diverse communities develop a gender identity different from the sex assigned at birth. Gender diverse youth are those whose sex assigned at birth is different to their felt sense of gender, this may be expressed through transitioning across the western gender binary, e.g. from man to woman. Identities such as; non-binary, agender, and genderqueer are characterized as being outside of the western essentialist gender binary of ‘man’ or ‘woman’ (Monro, 2005; Thorne et al., 2019). These communities face an increased risk of mental health difficulties hypothesized to be caused by systemic discrimination which is then internalized and may cause anxiety, depression, and suicidality (McLean, 2021; Meyer, 2003; Pearce et al., 2020).

Gender diverse communities have faced increased attention from media outlets (McLean, 2021), politics (Hines, 2020), and academic research (Coleman et al., 2022), locating them within a ‘culture war’ that questions the legitimacy of their identities (Skinner et al., 2024). There are claims made by trans-exclusionary radical feminists (TERFs) that online trans communities and peer influence provide a form of social contagion for young people to mistakenly decide they are trans (Littman, 2018; Pang et al., 2022). Such claims have been termed rapid onset gender dysphoria (ROGD) and have been used to justify restricting gender-affirming treatments as young people may later regret any interventions as they are not really trans (Ashley, 2020; Skinner et al., 2024). However, research has critiqued claims around ROGD and connection with online trans communities making people trans by highlighting various methodological concerns and issues with scientific rigor. For example, young trans people’s voices who are said to have ROGD are not represented in the literature and recruitment of participants through organizations that challenge the legitimacy of trans identities (Ashley, 2020; Restar, 2019).

Research has frequently looked at physical/transitional and mental health outcomes due to the difficulties with mental health and access to affirming care (Coleman et al., 2022), however, the role of online communities in formation of a gender diverse identity is still poorly understood. Although there is no definitive theory of gender diverse identity development, ‘milestones’ have been proposed for gender non-conforming youth from a largescale study of transgender diversity (Beemyn & Rankin, 2011; Rankin & Beemyn, 2012). Notably, some milestones were less applicable to some younger trans people who had more access to information, learnt about, and accepted being trans earlier in life. Many younger trans people knew about and had connected with other trans people earlier than older participants and the authors considered that the internet had facilitated such experiences. Additionally, a lack of information about and representation of non-binary identities has been found to impact identity development causing feelings of isolation and difficulties in developing a sense of wholeness within society (Cosgrove, 2020; Rankin & Beemyn, 2012). Although the importance of connection with gender diverse communities has been noted (Cosgrove, 2020; Ward & Lucas, 2023), with some suggestion that online spaces may facilitate this (Rankin & Beemyn, 2012), no research has specifically focused on the role of online communities and gender development for gender diverse young adults.

Craig and McInroy’s (2014) research on the internet and its implications for LGBTQIA + youth aimed to address the ‘phenomenon’ of young people ‘coming out’ earlier than in previous decades which was later explored further by Fox and Ralston (2016). Craig and McInroy (2014) found that online communities offer a safety net for the LGBTQIA + youth who use them. This safety net was comprised of; community resources not found offline, a place to find likeness and explore their identity for those who live in areas less visibly queer, and the ability to ‘come out’ online, before introducing this to the ‘real world’. The findings of this study influenced their future work which explored why online spaces are safer for gender diverse youth and found that online spaces provide a critical lifeline (Austin et al., 2020). These findings have been echoed by contemporary studies, and earlier research (Cavalcante, 2017; Evans et al., 2017; Jenzen, 2017; Selkie et al., 2019).

It is estimated that 91% of young people in the U.K. use the internet daily (Royal Society for Public Health, 2017). Using social media has been linked with increased access to language and representation for gender diverse youth, particularly those who are isolated, enabling a better understanding of their own identities (Goldberg & Kuvalanka, 2018; McInroy et al., 2019; Rankin & Beemyn, 2012). A recent quantitative study found that gender diverse adolescents (11–18 years old) referred to a Gender Identity Service in Germany used social media for finding specific information regarding gender exploration, however, the authors suggest that future research should explore how such online experiences impact gender identity development (Herrmann et al., 2024). How social media may be instrumental in developing identity could be explained through Uses and Gratifications Theory (UGT) (Ruggiero, 2000) and the Online Disinhibition Effect (ODE) (Suler, 2004). Both theories posit that the anonymity afforded by some online spaces leads to greater rates of disclosure (Clark-Gordon et al., 2019). UGT suggests that a persons’ use of the internet can be derived from two types of gratification: content, where a user benefits from the content of information—such as learning about transition and gender diversity, and process gratification—what a person benefits from utilizing the information, such as connection to people with shared identities. These are the two processes that drive a persons’ internet usage (Chen, 2011; Ruggiero, 2000). Evidence has been found to support the existence of UGT on modern social media platforms, such as Facebook, which uses content to drive engagement in groups and on a person’s Facebook feed (Ferris et al., 2021; Lin & Chu, 2021). The ODE, however, looks at how the anonymity afforded by the internet leads to greater rates of self-disclosure due to the feeling that since a person disclosing is not going to meet the person on the other screen, they are more likely to ask questions which they would not feel comfortable asking in the offline world (Suler, 2004). Reviews of evidence in 2012 and 2019 found that contextual factors relating to disclosure moderate the size of the ODE and support its existence as a form of increasing self-disclosures online (Clark-Gordon et al., 2019; Nguyen et al., 2012). This effect may be important in understanding the findings of McInroy et al. (2019) who reported that LGBTQIA + youth perceive online communities to be safer and better for seeking advice than offline communities.

## Research questions

The current research explored the relationships gender diverse young adults have with online LGBTQIA + communities. Specifically, what environment these spaces offered for the population to navigate their identities, as these communities may have an added importance in light of the trans healthcare service strain in England (Torjesen, 2018), and the growing hostility toward gender diversity in society (Hines, 2020; Pearce et al., 2020). The research also explored the gap in research on gender identity development online as gender diversity is underrepresented (Bradford et al., 2019; Diamond et al., 2011) by trying to understand from gender diverse young adults if these spaces have contributed to that developmental process and why they think that may be. To summarize, there are two research questions investigated within this study:

1. How do gender diverse young adults experience online spaces, as a “safe” environment?
2. Can online spaces facilitate gender identity development as suggested by McInroy and Craig (2018), and Craig and McInroy (2014)?

## Method

### Design

This study explored the experiences of gender diverse young adults using online LGBTQIA + spaces, using semi-structured interviews and was analyzed using Reflexive Thematic Analysis (RTA) (Braun & Clarke, 2022). The analysis made use of a deductive theoretical approach, as the study aimed to explore the experiences through applying existing theory. This research also used a post-structuralist feminist transgender theory to remove gender from a binary perspective and toward a gender pluralist theory which envisions gender and sex as a spectrum (Monro, 2005).

### Participants and recruitment

9 participants were recruited between 19–25 years of age. Further demographics are shown in Table 1 and language used is the participants’ own. Most of the participants were white, one participant chose to not have their video on during the interview and did not disclose their ethnicity in the demographics form. The participants had a range of gender identities and sexualities. Sample sizes within thematic analysis are a contentious topic with Braun and Clarke (2016) noting there is no true recommended sample size, as it depends on how rich the data collected will be for analysis.

**Table 1.** Participant demographics.

Pseudonym	Age	Gender Identity	Sexuality	Ethnicity	Pronouns
Amy	19	(Transgender) Female	Bisexual	White	She/Her
Ari	24	Non-Binary	Bisexual	White	She/They
Bob	20	Non-Binary	Gay	White	They/Them
Cam	19	Transmasculine Person	Asexual	–	He/They
D	25	Non-binary Transmasc	Asexual spectrum	White	They/He
K	20	Non-Binary	Pansexual	White	They/Them
Maria	19	Trans Female	Bi/Pan	White	She/They
TB	25	Male	Pan	White	He/Him
Vincent	22	Trans Male	Gay	White	They/Them & He/Him

Advertisements were placed on public social media platforms, e.g. Twitter, Tumblr, TikTok, for wide reach, and using the authors’ networks with the following inclusion criteria: are gender diverse, live in the U.K., aged between 18–25 years old, and who have/still engage with online LGBTQIA + spaces. Since gender diverse people use online spaces for community connection (Herrmann et al., 2024), and younger people are increasingly using social media (Royal Society for Public Health, 2017), recruiting online was considered an appropriate methodological design. However, the authors recognize that this recruitment method may have not reached participants who have had unsupportive experiences in online communities and are therefore avoiding social media platforms. All participants were from the U.K., except for one who signed the consent form, but disclosed in the interview they were from New Zealand. Digital copies of the participant information sheet, consent form, and demographic data form were sent to participants who showed interest. Individual online interviews took place using a university’s secure learning environment and averaged 48 min. Participants were not offered any incentives.

### Development of interview schedule

Semi-structured interviews were used due to their flexibility and being dependent on participant responses (Kallio et al., 2016; McLeod, 2012). The interview schedule was informed by Kallio et al.’s (2016) framework for creating interview schedules and relevant literature (Craig & McInroy, 2014; Fox & Ralston, 2016; McInroy & Craig, 2018) to guide the development of questions pertaining to identity and connection to online spaces. Interviews allow for narrative explanations of gender identity development and individual experiences to be heard (Bradford et al., 2019).

### Ethical considerations

Ethical approval for the study was gained from The University of Northampton’s Faculty Research Ethics Committee (FHSSOC000324) and the study followed the British Psychological Society’s (BPS) Ethical Guidelines (British Psychological Society, 2014). Informed consent was gained from participants electronically by signing a digital consent form and verbally before beginning the interviews. Further ethical consideration was given to conducting research on a sensitive group, the best efforts have been made to work ethically for the gender diverse communities as outlined by the BPS in their Sexual and Gender Minority guidelines (Barker et al., 2019). In addition, Vincent’s (2018) recommendations for ethical recruitment and collaboration with transgender participants in academic research were followed.

### Data analysis

The analysis was both deductive and constructionist, meaning that the analysis was directed by existing concepts and looks at how a certain reality is created by the data (Braun & Clarke, 2022). It drew upon influences from McInroy and Craig (2018) and Craig and McInroy (2014) as they developed the general theory underlying online sexual identity development but did not to consider how this may be different for gender diverse populations. Further inspiration was drawn from Fox and Ralston (2016) who suggest that feeling connected to the online space is beneficial for developing one’s identity and experiences of these online communities. The data was analyzed following the six phases of RTA (Braun & Clarke, 2022) as outlined in Table 2. Interview transcripts were split between the two authors for analysis. Regular meetings were held to discuss the analysis and review the development and write-up of the overall themes. The authors were in agreement of the overall themes.

**Table 2.** Reflexitve thematic analysis phases.

Steps	Worked example
Familiarisation	Reviewing the data through multiple readings to become immersed in the transcript and develop a deep understanding of the data.
Doing coding	Working systematically through the data and assigning a code label to aspects that speak to the research question. For example, “online safety”, “boundaries and protection”, and “virtual live vs real life”.
Generating initial themes	Exploring areas of the data for similarity of meaning and clustering together these codes into themes. For example, “online vs offline” and “online education spaces”.
Developing and reviewing themes	Reviewing the validity of the initial clustering from the previous stage and exploring possibilities for any further development of patterns of meaning.
Refining, defining and naming themes	Continued development and refinement of themes, involving structural and narrative work for the overall flow of the individual theme and overall analysis.
Writing matters for analysis	Deep refinement to translating the structure and narrative from the previous stage into a written analytic story of the whole data.

### Reflexivity

Both authors identify outside of the gender binary, as gay, and white British, and therefore share some similarities with the participants. One author has a Masters degree and is neurodiverse, the other has a Doctoral degree. The authors acknowledge their positions as affirming gender diversities, recognizing such experiences as part of human diversity. Being part of the study’s communities allowed the authors to engage with some of the nuances of the experiences described and avoid interpreting the participants’ experiences through a medicalised, cisgenderist, or heteronormative lens. Both authors have previously worked with gender diverse young adults in various capacities, including youth work, psychotherapeutically, and clinically. Therefore, the authors recognize the complexities and nuances of gender diverse experiences and are critical of how these have historically been simplified and pathologized by psychological professions, ultimately shaping perceptions of “natural” identity development.

## Results

### Theme 1: Safe spaces in virtual places

This theme focused on how the participants discussed how online communities are safer ways to express themselves. Participants talked about boundaries when interacting with online spaces and how they can help create a safe environment.

#### Theme 1A: Online versus offline

The participants experienced the safety of online LGBTQIA + spaces in multiple ways, for example, through comparison with offline spaces being historically dangerous, space for exploration of identities and management of “coming out”, and opportunities for conversations.

For Vincent, a 22-year-old trans male, there were significant differences in the online and offline spaces, with particular emphasis on aspects of safety: “The outside world is dangerous and historically we have been targeted in violent crimes. When considering this I think it is vital that people have access to these spaces”. Vincent described the offline world as “dangerous” and referred to how gender diverse people have been historically discriminated against, including violence. They used this understanding of the world as unsafe to highlight the significance of access to online LGBTQIA + spaces for gender diverse people to feel safe.

The safety provided by online spaces allowed for gender diverse people, such as Bob, a 20-year-old non-binary person, to “explore” their identities whilst maintaining a feeling of safety that is not commonly possible offline:

Someone in one of our group chats on Twitter – which isn’t LGBT groups – they haven’t actually come out to their parents yet, but on Twitter they can openly be a NB [non-binary] gay person, uhm, but irl [in real life] they still use she/her and pass as straight… It’s like, a chance for them to explore it without leaving the safety of their own little bubble.

The sense of safety that Bob discussed was made more possible through the online group as the person they mentioned could better manage their identities through exploring being openly non-binary and gay whilst “passing” as straight in “real life”. Bob highlighted the importance of being able to “come out” digitally, before expanding that version of self to the offline world in accordance with McInroy and Craig (2018) stage of coming out online, as it provides an increased sense of safety.

When comparing online and offline environments, participants, such as Vincent, talked more about how the comfort of living online allows them to have conversations they may be uncomfortable with having face-to-face:

I was able to talk about things that could have potentially been too embarrassing for me to bring up in face-to-face conversation. Not only this as many of us have to be closeted we don’t have that opportunity to have open face to face conversation about LGBTQI + issues.

Online spaces provided opportunities where the participants could discuss topics, such as, their identities, whilst being “closeted” offline. Additionally, Bob shared how the “anonymity of online spaces is sometimes better for talking about touchy subjects”. Vincent also shared a similar narrative: “this [anonymity] can be especially helpful when you are trying to work out what your gender is because you don’t have to worry about fitting into a certain category or molding yourself into a created persona online”. Online spaces helped mitigate feelings of “embarrassment” by provided more opportunities for conversations and navigation of identity partly due to the increased anonymity.

K, a 20-year-old non-binary person, provided a contrasting perspective to the other participants by drawing on the nuances of offline interactions:

I guess speaking to someone in person is totally different to talking to someone online, cause you can read emotions uhm so and like body language and that so they can actually see how you’re feeling when you’re, when you want the advice or when you’re giving it, they’re able to read you a lot better, and that way you’re able to connect to that person a lot better as well rather than online.

For K, being able to speak to someone offline allowed for a reciprocal process of both parties seeing and receiving non-verbal cues from body language, which helped a sense of comfortability and ability to connect.

Therefore, the participants recognized various components that were important to their sense of safety both online and offline. For some, the anonymity of online spaces aided their identity development through enabling engagement in conversations about their genders. Whereas, for others, there was a need for the embodied and proximity of offline interactions to feel understood.

#### Theme 1B: Online safety: boundaries and protection

The participants spoke of looking for online groups compatible with their expression, they would check for rules and/or moderation (typically found on a group page), and would specifically look for groups inclusive of gender diverse people (if browsing for a group based on sexuality). For example, Bob and Vincent only searched for online spaces which were inclusive of their non-binary identities: “I am always very careful to check that non-binary identities are recognized in any community I talk to.” (Vincent). Bob explains how they identified such inclusivity through clear rules set on the page, and how this led to feeling compatible with the group:

One thing I look for is the kind of inclusivity of GNC [gender non-conforming] people in pages that are centered around lesbians, because then it eliminates the kind of lesbians which are like you know “gold star lesbians are the only good ones” which is what I kind of want to avoid.

As non-binary people, it was important for Bob and Vincent to clearly see that the group was specifically inclusive of their own gender diversity. Through this process, Bob, highlights how they cultivated connections with communities of like-minded people as a self-protection strategy, i.e. connecting with and avoiding certain people. Maria, a 19-year-old trans female, also shared her experiences of being in quite negative spaces which affected her mental health and limited her growth and how she managed this:

I think it’s more like how to deal with a lot of negativity I think if someone’s giving you grief, being homo- trans- phobic etc it’s okay to just block them. And like a lot of the time on these spaces, you don’t have to interact with these horrible people you can just block them out and I think it’s healthier to block them out.

There is a suggestion that online spaces may require a certain amount of navigating and curating in order to be experienced as positive and that queerphobic people do exist in online LGBTQIA + spaces. Maria uses a method of blocking to create a “healthier” experience through surrounding herself with people who are affirming of their identity. Ari, a 24-year-old non-binary person, discussed a similar approach to limit interaction and exposure to discriminatory voices:

I’m just scrolling through my phone at the end of the day kind of thing, I don’t wanna see that all the time, there needs to be some kind of balance. The spaces I’m just engaging with as a person, I try and avoid that I guess.

The process of filtering out negativity helped improve some participants’ experiences on social media platforms and indicates some level of boundary regulation to derive both content and process gratification (as per UGT). For example, from being able to set privacy and safety related boundaries to engage and use media to increase their satisfaction. Where participants were not able to utilize boundaries within the groups, the purpose of engaging with the community shifted, as seen in Amy’s (19-year-old transgender female) experience:

It [Reddit group] was very much like this is a very insular group that doesn’t necessarily have the most real-world experience of being trans. It was a lot of people who were newly come out and we’re dealing with the fall out of that and I just kind of realised eventually that I wasn’t getting anything from being part of it. I was just exposing myself to other people’s trauma essentially and that was not the best thing for me mentally and also the amount of just petty drama that got built up… I kind of think of it as like it served a purpose almost like when I was first finding a community of other trans people, when I was first figuring things out was really helpful. But then once I had kind of grown beyond that it, I didn’t see it as a community anymore. I just saw it as people shouting into the void.

Participants were generally very aware of the management of online groups through rules and moderation, such as TB, a 25-year-old male: “I know obviously there is rules for the group to be respectful and no harassment and bullying and things”. Some participants, like Maria, were not as focused on the rules of the group in their initial approach to finding online spaces: “like I just join whatever, I see what it’s like and then in a few weeks or months, if it’s positive or negative, then it kind of depends on that”. Despite their different approaches, the participants jointly looked for “positive” and “inclusive” spaces, which were created through group rules and active moderation teams.

The moderation teams facilitated the safety of the online environment through monitoring posts against the rules of the group and managed replies to posts, to ensure overall inclusivity. For example, Bob spoke of post-checking: “admins who checks posts before we post, makes sure they’re not breaking any rules or offensive in any way” and Ari highlighted the how certain voices are managed:

Some of them [group members] are a group of admins and so there are rules however like if someone posts a question only TGNC [trans and gender non-conforming] people can reply for like 6 hours or something, uhm, so that the voices are actually heard rather than it being a free-for-all which is kind of cool.

Overall, the participants valued the role of the moderators and rules set and did not experience such management of the spaces as restrictive or limiting. Rather, having robust process in place with inclusivity and respect at core were recognized as positives, which helped to establish and maintain a sense of safety.

A final component of a positive group environment was reflected in activities which members could get involved with to bond and create a feeling of community, as illustrated by Maria:

Before lockdown and stuff we could you know meet up and stuff, but other times there’s challenges and hashtags and stuff going around, it’s [Discord] very similar to Twitter but there’s like the @everyone function, and announcements channel, so every now and then someone will @ everyone and inform them of stuff going on.

Maria talked about how community spaces facilitated good-natured events to keep engagement and positivity within the space. For example, they opened dialogues, facilitated new friendships, and keep all users up to date with any events. Activities like these helped to create a feeling of togetherness and improve bonding between members.

In conclusion, participants spoke of mixed experiences using online LGBTQIA + spaces, which included increased anonymity to have more sensitive conversations and identity management by being able to explore their gender diversities online whilst being selectively “out” offline. However, for some, there was a need to manage the online spaces through enforcing boundaries and curating who they were connected with. Finally, participants sought spaces which were explicitly inclusive in their group rules and appreciated management of the groups *via* the rules and moderation teams, which created a sense of safety, centered diverse voices, and abled a sense of community.

### Theme 2: Value of online spaces as educational resources

This theme presents evidence that was found to support multiple aspects of identity development, as identified by Craig and McInroy (2014). Participants spoke of gaining access to resources they would not find elsewhere else as this knowledge is created by the communities for the communities, a way to explore their gender identity safely, find likeness in the expression of others, accept their identity, and engage in disclosure online. Online spaces were also invaluable for coming to terms with their own gender identities, with some participants, such as D, a 25-year-old non-binary transmasc person, suggesting they would not have known their gender identities without them:

I definitely would not be the person I am today without those spaces. When I first joined, I thought I was still cisgender. I learned about transgender people and kind of within a year of that was seeking out a gender identity service to start talking about the options I had for transitioning.

For D, online spaces provided information to learn about gender diversity, which ultimately resonated with them and enabled them to start the process of transitioning. D’s experience suggests that the information about gender diversity was not available or easily accessible offline. This experience was also shared by other participants, such as Cam, a 19-year-old transmasculine person:

There was no education in school, there was no information, I went through a kind of activism kick for a bit and wanted to understand all the people. So, I started looking around online to try and understand others and then ended up identifying with some labels a lot more than I thought I would.

Normative spaces for learning, such as schools, did not provide the participants with inclusive information about gender, meaning a common experience was for them to turn to online spaces to seek out their own knowledge. Through this process, online spaces allowed participants to gain the information they had been lacking. In particular, TB spoke about the varied representation online and how he saw that transitioning “can take a while” and that “not everybody automatically changes overnight”. TB also shared how the online groups were also useful for seeing people who were “all at different stages of their transition”, highlighting the process of transition that was made accessible through the online spaces.

Additionally, participants, such as Amy, spoke about the importance of the online spaces for early stages of gender exploration:

It offers people to talk to about problems that you’re facing. It offers companionship and knowing that you’re not alone early on. It can offer practical things like advice on how to get started on HRT or any practical advice really and it can in some senses, in some situations, offer friendship.

The groups offered a sense of “companionship” and “friendship” that enabled Amy to not feel alone and provided a space to gain the practical advice which Cam said was lacking in schools. In addition to providing information and practical advice, the spaces also allowed for participants like D to create their own knowledge, as they were connected with people with similar experiences:

I mentioned that I’m in transmasc group on Tumblr, and over the past two years we have been in a position where we are trying to actually invent new language to talk about transmasc experiences. I was actually part of one of the groups that invented a word.

These quotes exemplify the nature of online LGBTQIA + communities’ power to make (and create) knowledge of diverse identities accessible allowing people to explore their genders in an environment where all expressions are validated. Such spaces directly facilitated the interviewee’s understanding of gender and allowed Bob to acknowledge what “had been there the whole time”:

Compared to like 2 or 3 years ago, my knowledge base, and my opinions are completely different, because of the amount of education these people have given me and the support these people have given me.

Bob continued to talk about the personal growth they had since accessing these spaces, further highlighting their importance as educational resource and form of support to facilitate development.

To conclude, the participants spoke of the lack of education and representation of gender diversity which facilitated a lack of awareness of their own identities. Many participants turned to online groups in search of information, which were a key turning point in their identity development, as the groups provided the information that offline spaces, such as schools, lacked. This enabled the participants to gain representative information, practical advice, create their own knowledge, and form friendships that ultimately shifted opinions about themselves, allowing for connection with their sense of gendered self.

## Discussion

This study explored how gender diverse young adults engaged with online LGBTQIA + communities to aid their gender identity development, and what these spaces offered that offline communities cannot. Participants spoke of how these communities are structured, how they offered invaluable educational resources, and how they came to understand what gender meant to themselves through this engagement. The analysis suggests LGBTQIA + online spaces are vital resources with positive effects for developing a congruent gender identity. All participants made clear that without access to these spaces their gender identity would not have been known. This suggests the nature of these groups being affirmative and making non-binary identities visible is important to the formation of diverse gender identities that are outside the essentialist western gender binary (Nagoshi et al., 2014), and current models of transgender healthcare (Torjesen, 2018; Wagner et al., 2019). Participants also noted the importance of these spaces as educational resources for development. The communities have created their own knowledge that cannot be found elsewhere due to a lack of understanding of transgender specific issues in educational and healthcare contexts. Medical transitions remain inaccessible in the U.K. for a large population due to ever-growing waiting lists, and a lack of general practitioner knowledge (Torjesen, 2018; Whitehead, 2017). This evidence underscores the importance of being able to access resources for these communities, in line with current research on online LGBTQIA + communities for sexual minority youth (DiFulvio, 2011; Fox & Ralston, 2016; Russell & Fish, 2016).

Online spaces can be perceived as a safe space through responsible moderation practices, such as having clear and fair enforcement of the rules, members of the administrative team also had to have a positive presence on the space to be taken both seriously and be respected (Hetrick et al., 2016; Seering et al., 2019; Squirrell, 2019). Online spaces were found for some participants to be better to have “embarrassing” conversations through the ODE and having a sense of anonymity for closeted members that one would not get in an offline space (Cipolletta et al., 2017; Craig & McInroy, 2014; Hollenbaugh & Everett, 2013; Suler, 2004). Participants highlighted the importance of enforcing their own boundaries and curating their online experience where moderation practices were not present or effective, such, as, being able to unfollow, unfriend, and block sensitive topics (Frederic & Woodrow, 2012). Filtering out negativity has been shown to improve ones’ experience on social media platforms, and one of the biggest selling points of modern social media—as opposed to internet forums (Barnidge et al., 2019; Bode, 2016). Where participants were not able to enforce boundaries within the online spaces, the spaces were experienced as unhelpful, and they eventually left. The research found consistent criteria between some participants of what had to be present to join a community: compatibility, clear rules and enforcement, and inclusivity of identities outside the mainstream LGBTQIA + communities. This tripartite explanation also fits with UGT whereby the way these gender diverse people engage with these spaces to derive gratification is linked to their safety online (Chen, 2011; McInroy et al., 2019a). These three items summarize an issue for gender diverse people within society currently; that it is inherently unsafe due to rising hate crime and a mainstream adoption of exclusionary essentialist views (Pearce et al., 2020; Taylor et al., 2019). The safety of these spaces then allows gender diverse people to become visible, affirmed, and recognized for who they are when there is no legal recognition of non-binary identities in the U.K. (Goldberg & Kuvalanka, 2018).

## Implications and limitations

Given the growing importance of online communities and increasing rates of social media usage (Royal Society for Public Health, 2017) future research would benefit from investigating further how the interactions with these spaces through a UGT and ODE framework foster this developmental process for gender diverse youth, and what may further positively influence this development. Implications for practice include healthcare providers and practitioners working with gender diverse people addressing the imbalances in provision of gender affirming healthcare by exploring how some of the benefits for identity development found in the online communities can be embedded in the services they provide. Additionally, future research is needed to explore how educators can help connect gender diverse young adults to each other both in-person and *via* online communities to foster identity development.

The authors recognize that safety is contextual and therefore the current research only reflects representations and understandings from a small sample of participants who were majority white. Further marginalized identities alongside gender diversity may complicate the feeling of safety in online spaces and impact identity development, for example, due to racial discrimination (Cyrus, 2017). This would likely present different accounts to the one’s articulated by the participants in the current research and is therefore an area for further exploration. Additionally, recruiting participants online may have limited the sample and experiences heard, as young adults who have had unsupportive and/or non-affirming encounters with LGBTQIA + online communities may now avoid these spaces and therefore would be less likely to see the recruitment poster.

## Conclusion

This research highlights the importance of gender diverse young adults accessing online LGBTQIA+ communities to build a sense of identity through finding people who are similar, community belonging, and improving access to specific resources relating to gender diverse issues. The research also shows consistency with other narrative analyses of gender development, whereby participants all developed their identity after engaging with dedicated spaces with access to resources and exposure to those with similar identities. These parts of online communities are shown to aid in identity development. Future research should focus on how gender affirming healthcare and practitioners working with gender diverse young adults can integrate the benefits of online communities to support them with their gender identity development.

## Data Availability

Due to ethical issues, data underpinning this publication cannot be made openly available. Further information about the data and conditions for access are available from https://doi.org/10.24339/79cfffd8-365e-42ff-89e9-9702624969c8

## Rights retention statement

For the purpose of open access, the author (s) has applied a Creative Commons Attribution (CC BY) license to any Author Accepted Manuscript version arising.
